# Contrasting trends in light pollution across Europe based on satellite observed night time lights

**DOI:** 10.1038/srep03789

**Published:** 2014-01-21

**Authors:** Jonathan Bennie, Thomas W. Davies, James P. Duffy, Richard Inger, Kevin J. Gaston

**Affiliations:** 1 Environment and Sustainability Institute, University of Exeter, Penryn, Cornwall, UK TR10 9EZ

## Abstract

Since the 1970s nighttime satellite images of the Earth from space have provided a striking illustration of the extent of artificial light. Meanwhile, growing awareness of adverse impacts of artificial light at night on scientific astronomy, human health, ecological processes and aesthetic enjoyment of the night sky has led to recognition of light pollution as a significant global environmental issue. Links between economic activity, population growth and artificial light are well documented in rapidly developing regions. Applying a novel method to analysis of satellite images of European nighttime lights over 15 years, we show that while the continental trend is towards increasing brightness, some economically developed regions show more complex patterns with large areas decreasing in observed brightness over this period. This highlights that opportunities exist to constrain and even reduce the environmental impact of artificial light pollution while delivering cost and energy-saving benefits.

During the 20^th^ Century the development of electric lighting and rapid growth of human settlement, transport infrastructure and economic activity has led to large areas of the globe coming under the influence of artificial light at night. This is due both to direct illumination and “skyglow”, the diffuse scattering of light in the atmosphere. In 2001, 19% of the global land surface was estimated to be above a threshold of light set for polluted status, and 21% of the world's people to live in areas where light pollution obscures views of the Milky Way^1^. Longstanding concerns about the implications for scientific astronomy and aesthetic enjoyment of the night sky^2^ have been joined in recent years by growing awareness of potential effects of artificial light on human health, ecological processes and ecosystem services^3^,^4^,^5^,^6^,^7^,^8^,^9^.

The marked increase in the intensity of atmospheric light pollution is evident from long-term observatory records^10^ and estimates of its spatial extent through analysis of satellite images^1^. While such images have been instrumental in raising awareness of the global spread of artificial light, there is however considerable potential for detailed, quantitative assessment of changes in the distribution and intensity of artificial light in the landscape. This is critical as patterns of change in nighttime lighting are becoming increasingly complex, particularly in industrially developed regions. On the one hand, increasing populations, urban expansion, economic development and more efficient lighting technologies continue to drive further increases in lighting. On the other hand, the global financial crisis of 2007/2008 and concerns over greenhouse gas emissions and energy security provide strong incentives to reduce the intensity and duration of public and private lighting, while technological developments allow systems to be more tightly controlled and offer design opportunities to minimise unnecessary trespass of light into the environment^7^. Meanwhile, in many developed countries the decline in primary industries and movement towards an information economy has reduced the need for lighting connected with industrial processes. As these trends develop, there is a clear need for tools to monitor and assess changes in light pollution across spatial scales, from the effects of a single urban or industrial development to national and continental trends. Here, we apply a novel intercalibration method to satellite images to detect regions in Europe that experienced either marked increases or decreases in detected brightness from space, as a first step towards detecting trends in light pollution across the continent.

A long time-series, global data set of annual nighttime satellite images from the Defense Meteorological Satellite Program (DMSP) operational linescan system (OLS) is publicly available and digitally archived from 1992, produced and provided by the NOAA National Geophysical Data Center^11^. The distribution of artificial light from these images has been used as a proxy for urbanisation^12^,^13^, population density^14^,^15^, economic activity^16^,^17^ and armed conflict^18^,^19^, as well as to assess the spatial extent of light pollution itself^1^,^20^,^21^. Quantifying changes in the brightness of light sources over time has been hindered by the lack of onboard intercalibration between satellite-borne sensors, and the fact that the gain control of the optical instrument is constantly adjusted to provide consistent imagery of cloud under changing moonlight conditions. For this reason making direct comparisons of the brightness of artificial lighting between years is problematic. Nevertheless, intercalibrations of the data set within an assumed “no change” region have been used to quantify change^22^,^23^, and recent approaches have used principal component analysis^24^ and robust regression techniques^13^ to overcome these problems and map changes in lighting in rapidly developing regions of Asia.

We created intercalibrated images of nighttime lights in Europe using quantile regression on the median, a robust regression technique not previously applied to this dataset, to intercalibrate DMSP/OLS satellite images. The advantage of this method of intercalibration is that it is insensitive to localised changes in brightness in the calibration set, and so strict assumptions of a “no change” region may be relaxed. We assessed changes in artificial lighting in terms of the extent of the areas decreasing and increasing in brightness over the region. The method was validated by the successful attribution of regions of both increasing and decreasing intensity in a calibration area in South-West England to urban and industrial developments, confirming that the observed direction and timing of change is consistent with known changes in nighttime light intensity on the ground. We then extended the approach to map areas of increasing and decreasing brightness across Europe. While the brightness of nighttime light pollution across Europe is increasing overall, clear regional differences exist, with considerable regions experiencing apparent net dimming over the period.

## Results

### Change map validation

**Figure 1:**
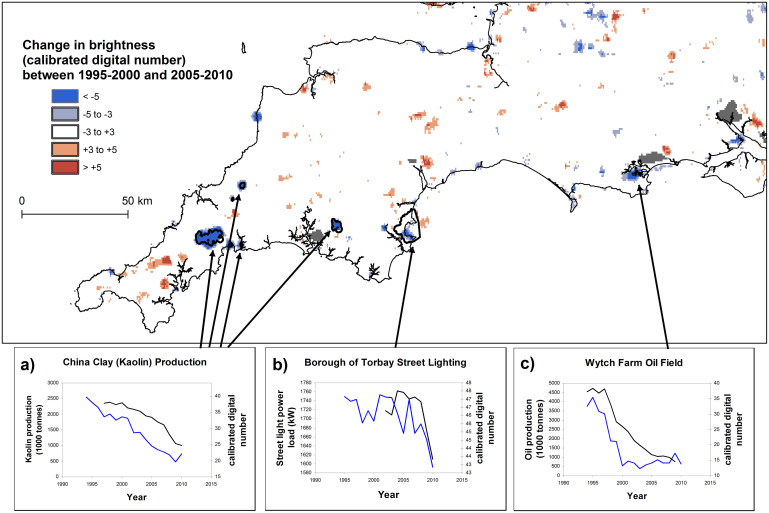
15-year changes in nighttime brightness in South-West England. Highlighted regions: (a) Annual trend in brightness for areas associated with the china-clay (kaolin) industry, (blue line); total china clay production (black line). (b) Annual trend in brightness for the urban region of Torbay (blue); total power load on municipal street lighting in Torbay (black). (c) Annual trend in brightness for Wytch Farm onshore oil field (blue); total oil production from the field (black). Map generated using ESRI ArcMap 9.2.

Within our calibration region of South-West England (Figure 1), change maps show patches of both increasing and decreasing brightness between 1995–2000 and 2005–2010. In the four predominantly rural administrative counties that comprise most of the calibration area, 98 discrete patches were identified that were apparently decreasing in brightness (<−3 Digital Number; DN) and 111 patches that were increasing in brightness (>+3 DN). Increases in brightness in this region were largely attributable to urban expansion, particularly new development of suburban residential and light industry (45.9% of patches, 64.4% of total increasing area), as well as agricultural and small-scale rural industrial buildings (33.3% of patches, 11.4% of area) or large industrial developments (15.3% of patches, 21.9% of area). Only six of the 111 patches (5.4%) that showed apparent increases in brightness in the region could not be attributed to a feature known to have increased in brightness over this period. Apparent decreases in brightness were predominantly attributed to areas associated with mineral extraction industries where production declined or ceased (15.3% of decreasing patches, 50.8% of decreasing area), existing small urban areas in which street lighting infrastructure was modernised during the period (44.9% of patches, 21.7% of area), and military or other government infrastructure which was closed or redeveloped (7.1% of patches, 13.2% of area). Six patches (6.2%) could not be attributed to a feature likely to have declined in brightness. Examples of patches of declining brightness are highlighted in Figure 1, including infrastructure associated with the china clay (kaolin) industry (quarries, processing infrastructure and ports), and the Wytch Farm oil field, which have both undergone significant declines in production alongside the decline in observed brightness. The urban area of Torbay is also shown. Here the majority of the existing stock of high-pressure sodium lighting fixtures was modernised and replaced with more efficient units during the years 2008 and 2009, causing a sharp decrease in both energy use and detected brightness.

### Changes in European light pollution

**Figure 2:**
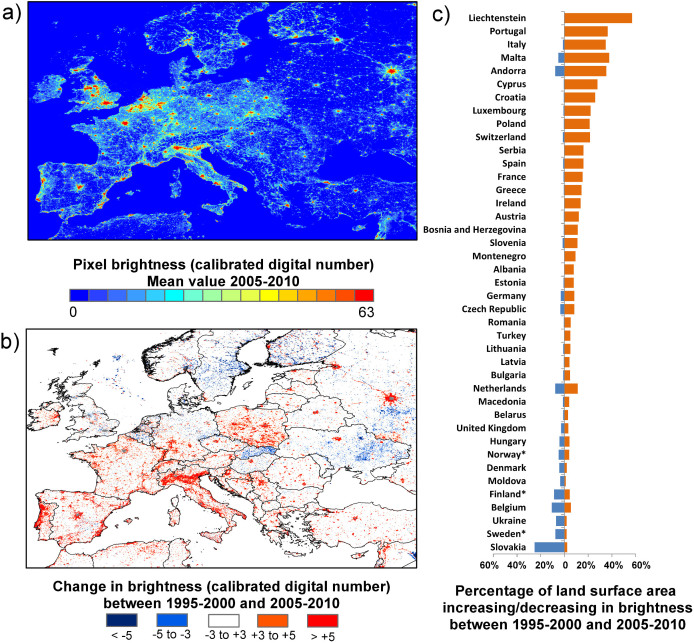
(a) Intercalibrated mean brightness for Europe 2005–2010. (b) 10-year change in brightness, calculated as the difference in mean values for the periods 2005–2010 and 1995–2000. Grey areas are saturated throughout the time period, so trends cannot be detected. (c) Proportions of the total land surface area for which artificial light was detected to increase (orange) and decrease (blue) by more than 3 DN units in constituent countries of Europe. *Data south of 65 degrees latitude only. Map generated using ESRI ArcMap 9.2.

In common with recent studies in Asia^13^,^16^,^24^, Europe has experienced a marked net increase in nighttime light pollution since satellite images first became available (Figure 2). Inferences about heavily urbanised areas must be treated with caution as the DMSP/OLS sensors saturate at high light levels; however, marked regional differences within the unsaturated rural and suburban areas exist. It has been previously noted that large areas of some countries of the former Soviet Union, such as Moldova and Ukraine, experienced a contraction in lighting following independence^22^; the effects of this change are still evident in this study over a more extended time period. Widespread decreases in brightness also occur in Hungary and Slovakia. Moreover, we find that several economically developed countries, including Sweden, Finland, Denmark, Norway, the United Kingdom, Belgium and Northern Germany also show areas apparently experiencing detectable localised declines in brightness.

## Discussion

**Figure 3:**
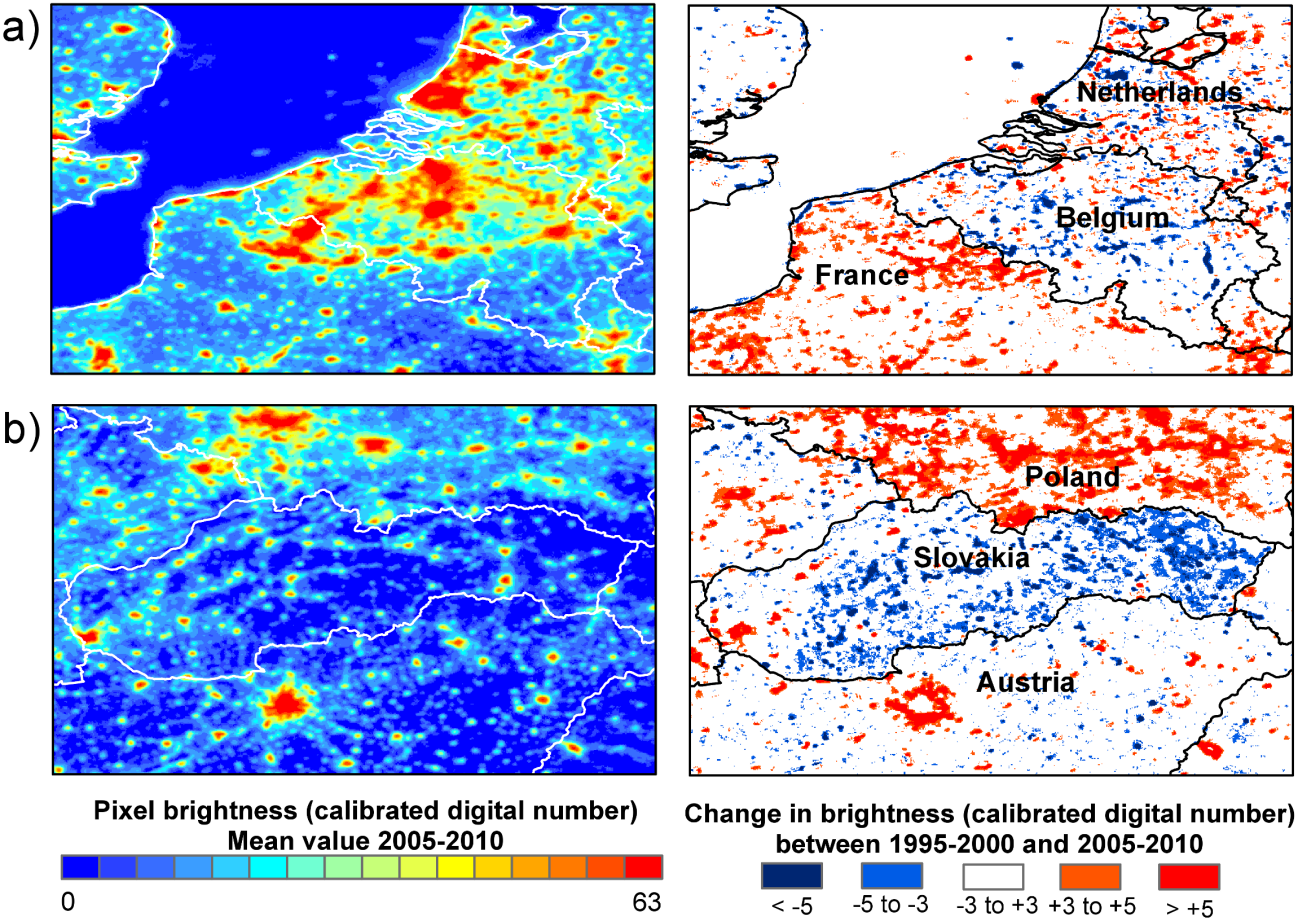
Selected areas of maps shown in Figure 2, showing contrasts in trends in detected nighttime light between different countries. (a) Belgium shows decreases in nighttime brightness along the motorway network, while neighbouring regions of France have increased substantially in brightness. (b) Slovakia shows marked decreases in brightness, with the exception of Bratslava and towns in the west of the country. In contrast, neighbouring regions of Poland have become substantially brighter. Map generated using ESRI ArcMap 9.2.

This study shows that regionally significant decreases, as well as widespread increases, in the brightness of nighttime lights are occurring across Europe. In part these may be attributable to economic or industrial decline, as has been noted for some countries of the former Soviet Union and Eastern Europe^22^, and the decline of sectors of the economy, as shown here with mineral extraction in South-West England. However, notable decreases are detectable in Scandinavia and little net change is detected in many regions of North-Western Europe, regions not associated with widespread economic decline during this period. Examination of the spatial pattern of change can provide insights into the causes of changes in brightness. For example, in Slovakia, for which our data shows widespread decreases in observed brightness across small towns and villages (Figure 3), many municipalities began switching off public lighting for part or all of the night for financial reasons during this period^25^; the capital Bratislava and several towns that underwent renovation of their lighting systems do not show an equivalent decrease^25^. In Belgium, which shows the second largest proportional extent of dimming, decreases in brightness are almost entirely associated with the road network, while towns and cities have tended to increases in brightness (Figure 3). Belgium is unique in Europe in having lighting installations for almost the entire length of its motorway system; during the time period covered by this study lighting in the central reservations of many motorways were switched off for environmental and financial reasons for periods during the night^27^. It is likely that the impacts of the global financial crisis of 2007/2008 will lead to more widespread stabilisation or reduction in light emissions in the developed world, although rapidly developing regions are likely to continue to increase their lit areas. In our calibration region, we have evidence linking net decreases in brightness to both declines in extractive industry and increased efficiency and energy use of municipal lighting. We are therefore able to identify those locations where reductions in light emissions have been achieved in combination with energy and cost efficiency benefits, and without excessive costs to living standards, rather than as a by-product of economic decline.

Nighttime remote sensing applications are likely to develop rapidly as higher resolution nighttime lights data become available, for example (as of 2012) from the Suomi NPP Polar orbiting satellite^28^,^29^. However, the DMSP/OLS data are currently unique in their temporal coverage and hence ability to track trends in light pollution over time. A certain amount of caution must be taken in interpreting apparent increases and decreases in brightness from these data as changes in absolute irradiance for several reasons. First, a single sensor channel cannot fully capture the multispectral irradiance of light sources. The spectral response of the OLS instrument differs somewhat from that of human or animal vision, or the action spectra of physiological responses – since it has a peak response at wavelengths between around 500 and 800 nm, changes to lighting types may misleadingly appear as decreases in brightness. Second, the links between artificial light observed from space and the environmental, health, scientific and aesthetic impacts of light pollution are complex; whilst imagery based on the emission of light upwards is the best available on global or large regional scales it may not always correlate well with direct illumination of the environment or horizontal emissions that cause skyglow. Finally, variation between and within measurements taken by different sensors due to the lack of cross-sensor calibration, atmospheric and surface conditions and ‘on-the-fly' adjustment of instrument gain mean that an absolute calibration of the DMSP/OLS nighttime lights images to irradiance is probably not possible. However, notwithstanding these difficulties in quantifying and interpreting changes in brightness on the ground, we show that with appropriate intercalibration these data can provide estimates of the direction and timing of changes in nighttime lighting that are highly consistent with other data sources. Furthermore, the resolution of the data is sufficient for individual decisions at the scale of an urban or industrial development to be evaluated, and global coverage enables detection of emerging trends at regional and national scales. There is a growing need to evaluate the effects of light pollution on the natural and human environment, and technological developments, along with growing pressure for energy efficiency and economic cost savings, provide opportunities to introduce measures to reduce the impact of nighttime light^7^. These measures range from simple low-tech solutions (such as focussing lighting only where it is needed, shielding sources from emitting light in a horizontal or upwards direction, removing or switching off lights when and where they are not needed) to high-tech (for example, adaptive lighting that dims according to weather, traffic conditions, moonlight or time of day). Careful analysis of satellite imagery can inform decisions on the need for, and efficacy of such measures, and help to identify how local economic and environmental decisions influence our global night environment.

## Methods

### Data source

Each DMSP/OLS image represents a composite of cloud-free images taken throughout the year during the hours of darkness. The data are nominally at 1 km resolution, resampled from data at 2.7 km resolution, and each pixel is represented by a digital number (DN) between zero and 63. A value of zero represents relative darkness, while very brightly lit urban areas typically saturate at values of 63. No onboard calibration of the sensor exists, and the time series includes data from six different satellites with different sensors, so the brightness of images must be cross-calibrated carefully in order to assess any change in brightness^11^. Furthermore, errors in geolocation for any given region result in a slight offset of up to 3 pixels (<3 km) in the apparent position of permanent features such as towns and cities between some years. To correct for geolocation errors each image was consecutively shifted by between −5 and 5 pixels in both the x (longitude) and y (latitude) directions and the Pearson correlation coefficient of all pixels with the corresponding pixels of the previous image was calculated. The x and y offset combinations with the maximum correlation of all 121 comparisons were recorded and the coordinates of each image adjusted accordingly to maximise the match of spatial pattern between images.

### Intercalibration region

Calibration between years using ordinary least-squares regression (OLS) in so-called “no change” areas, which are assumed to have had negligible changes in lighting throughout the period^23^, may be problematic as, for a full calibration, the area selected must contain the full range of DN values from completely dark rural pixels to saturated urban pixels, which must be lit at a constant brightness throughout the period. Arguably, few, if any, areas with this range of values can be reliably assumed to have had no significant changes in brightness over the past decades. Since the coefficients of ordinary least-squares regression are heavily influenced by outlying values, any pixels with real directional changes in brightness within the calibration region will influence the calibration and may lead to misleading patterns of change. Recent approaches^13^ have used a robust regression technique that iteratively removes “suspect” outliers to overcome this problem. We use an alternative robust regression technique for intercalibration, quantile regression through the median. This method differs from least squares regression techniques in that it estimates the conditional median, rather than the mean, value of the response variable given the predictor variable. The method is therefore inherently insensitive to outlying values^30^. The breakdown point, *ie*. the proportion of observations that can be arbitrarily high or low, of estimates of the median is 0.5. In this context this means that up to 50% of pixels with a given DN in a calibration image may have undergone a real increase or decrease in actual brightness without strongly influencing the estimate of the calibration coefficients. This makes it suitable for use in intercalibration in regions where limited directional changes in lighting are occurring over time, as instead of assuming that all pixels have a constant brightness over the entire calibration period, against which the measured DN may be intercalibrated, we only need to assume that at least approximately 50% of pixels have constant brightness. We selected a calibration region of 150 by 350 km covering south-western England (Figure 1). Here the majority of lighting sources are associated with fixed infrastructure such as municipal street lighting. Due to planning regulations in the UK, urban and industrial growth in the region is almost entirely located in discrete locations occupying a small proportion of the land surface; while there have undoubtedly been changes in lighting due to the increase in population, urban and suburban development, industrial development and decline, leisure facilities and changes in street lighting type and management, these changes are almost certainly localised in extent during this period and affect a minority of pixels. We therefore assume that most changes in brightness are highly localised in space (i.e. affecting a minority of pixels in any one year) and/or time (i.e. occurring during a minority of years for any one pixel).

Following correction for x- and y-shift, we intercalibrated images using 6^th^ order polynomial quantile regression on the median, using the package ‘quantreg' version 4.76 (http://CRAN.R-project.org/package=quantreg) in the statistical software R version 2.13 (R Foundation for Statistical Computing. Vienna, Austria. http://www.R-project.org). The year 1994 was chosen as a base reference, as the image had the highest proportion of pixels with DNs of both zero and 63; by intercalibrating to this year no subsequent year's image was extended beyond the range between the minimum detectable signal and saturation. Each consecutive year *t* from 1995 to 2010 was intercalibrated against this base year, by fitting the regression: 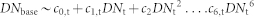
Where *DN*_base_ is the digital number of the pixel in the base year (1994), *DN*_t_ is the digital number of the pixel in year *t*, and *c*_0,t_ *c*_1,t_ … *c*_6,t_ are a set of six fitted regression constants used in converting raw digital numbers to a number intercalibrated against the base year. Values below zero and above 63 were truncated to zero and 63 respectively. Average values for regions of interest were extracted using ArcGIS v9.2. In those years when two DMSP satellites were operating simultaneously, data from the most recent satellite was used in the final analysis. To assess the effectiveness of the intercalibration method in standardising the scales of measurement, we compared calibrations using both satellites for these years. This analysis shows that the method is effective in removing the systematic bias in data due to different satellites (Supplementary Fig. S1 and Table 1 online).

### Assessment of observed direction and timing of change

To assess the ability of the methodology correctly to identify regions undergoing changes in brightness, areas with increasing or decreasing brightness were matched to features on the ground using visual inspection of maps and aerial images from Google Earth, and probable causes of change were attributed. A threshold of change of +/− 3 DN units was selected to identify discrete patches of contiguous pixels with a significant change in brightness over the period. Mineral production figures shown in figure 1 were supplied by British Geological Survey; Torbay street lighting data were supplied by Western Power Distribution.

## Author Contributions

J.B., T.W.D. and K.J.G. devised this study, J.B., T.W.D., J.D. and R.I. contributed towards the analysis, J.B. wrote the first manuscript draft and all authors contributed to revisions.

## Supplementary Material

Supplementary Information Supplementary figure S1 and table S2
