# Supplementary material for: Contrasting trends in light pollution across Europe based on satellite observed night time lights
Source: Sci Rep. 2014 Jan 21;4:3789. doi: 10.1038/srep03789 (PMC3896907; doi:10.1038/srep03789)
Supplement: Supplementary Information — Supplementary figure S1 and table S2 [file srep03789-s1.pdf]

Contrasting trends in light pollution across Europe based on satellite observed night time lights

Jonathan Bennie*, Thomas W. Davies, James P. Duffy, Richard Inger and Kevin J. Gaston

Supplementary Information

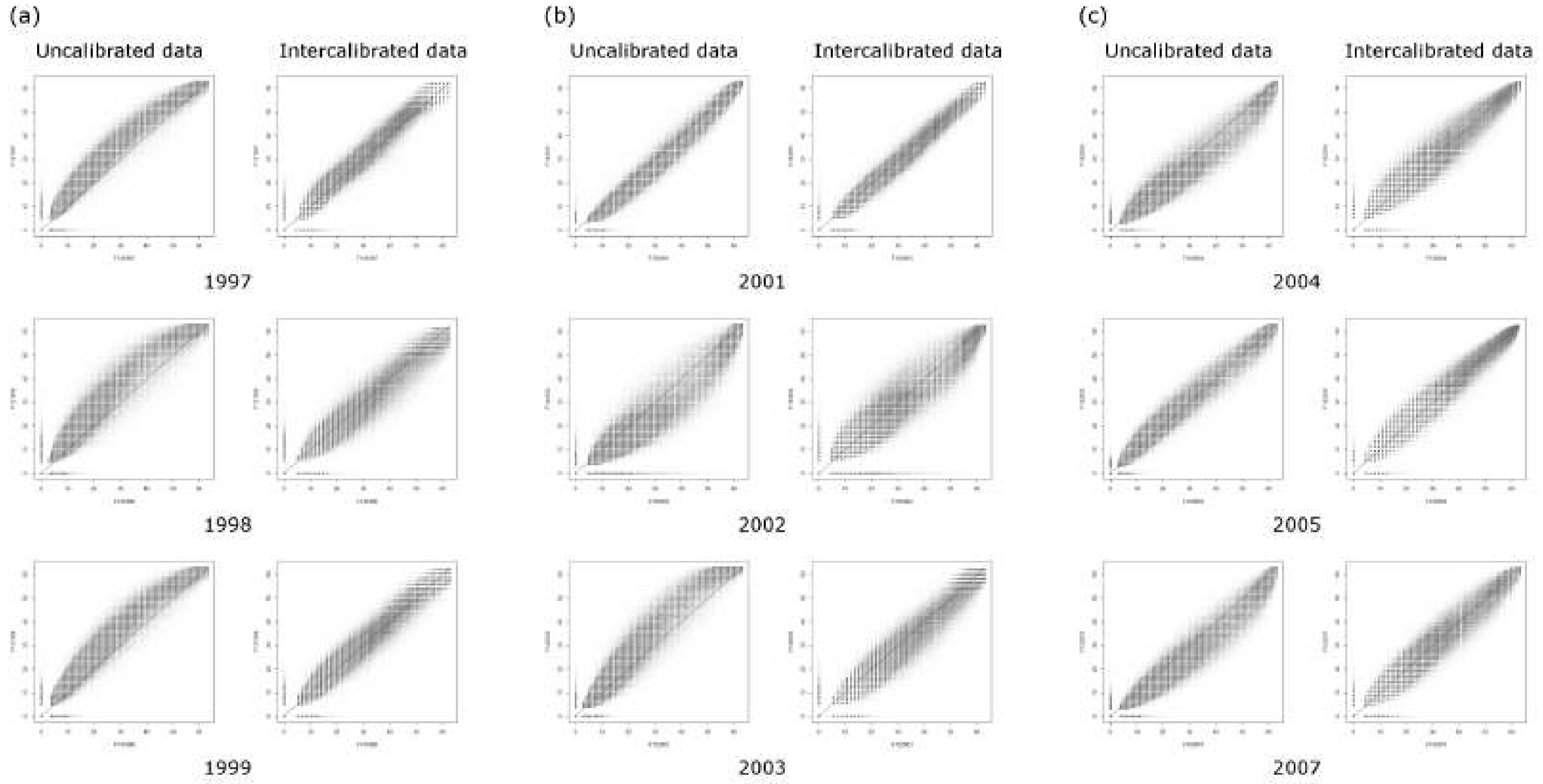

Supplementary figure S1: Scatter plots comparing nighttime light data for the same year but from different satellites for years in which two DMSP satellites were operational. (a) satellite F14 (x-axis) against F12 (y-axis); (b) satellite F15 (x-axis) against F14 (y-axis); (c) satellite F16 (x-axis) against F15 (y-axis). In each case the intercalibrated data increases provides a more linear fit and increases the consistency between datasets.

Supplementary table S1: comparison of mean error statistic for nighttime light data for the same year but from different satellites for years in which two DMSP satellites were operational.

Year	Satellite comparison	Uncalibrated mean error	Calibrated mean error
1997	F12,F14	1.04	0.30
1998	F12,F14	1.00	0.03
1999	F12,F14	0.74	-0.07
2001	F14,F15	-0.53	-0.21
2002	F14,F15	-0.87	-0.26
2003	F14,F15	0.62	-0.32
2004	F15,F16	-0.52	0.12
2005	F15,F16	0.05	-0.01
2007	F15,F16	-0.62	-0.04
